# Genetic and epigenetic background of diabetic kidney disease

**DOI:** 10.3389/fendo.2023.1163001

**Published:** 2023-05-30

**Authors:** Niina Sandholm, Emma H. Dahlström, Per-Henrik Groop

**Affiliations:** ^1^ Folkhälsan Institute of Genetics, Folkhälsan Research Center, Helsinki, Finland; ^2^ Department of Nephrology, University of Helsinki and Helsinki University Hospital, Helsinki, Finland; ^3^ Research Program for Clinical and Molecular Metabolism, Faculty of Medicine, University of Helsinki, Helsinki, Finland; ^4^ Department of Diabetes, Central Clinical School, Monash University, Melbourne, VIC, Australia

**Keywords:** diabetic kidney disease, kidney failure, GWAS, genome sequencing, exome sequencing, epigenetics, epigenome-wide association study, EWAS

## Abstract

Diabetic kidney disease (DKD) is a severe diabetic complication that affects up to half of the individuals with diabetes. Elevated blood glucose levels are a key underlying cause of DKD, but DKD is a complex multifactorial disease, which takes years to develop. Family studies have shown that inherited factors also contribute to the risk of the disease. During the last decade, genome-wide association studies (GWASs) have emerged as a powerful tool to identify genetic risk factors for DKD. In recent years, the GWASs have acquired larger number of participants, leading to increased statistical power to detect more genetic risk factors. In addition, whole-exome and whole-genome sequencing studies are emerging, aiming to identify rare genetic risk factors for DKD, as well as epigenome-wide association studies, investigating DNA methylation in relation to DKD. This article aims to review the identified genetic and epigenetic risk factors for DKD.

## 1 Introduction

A total of 537 million people worldwide have diabetes (1), characterized by elevated blood glucose. Despite treatment, which aims to normalize the blood glucose concentrations, diabetes can lead to micro- and macrovascular organ damage through various molecular pathways, including increased reactive oxygen species, which further affect the downstream pathways such as the polyol pathway flux, advanced glycation end-product formation and activation, protein kinase C activation, and the hexosamine pathway flux (2). These microvascular complications include diabetic kidney disease (DKD), sight-threatening proliferative diabetic retinopathy, and diabetic neuropathy. The complications reduce the quality of life, increase mortality, and account for the majority of the health care costs for diabetes (3, 4). Together, 30%–50% of individuals with diabetes develop DKD (5–7). Individuals with type 1 diabetes (T1D) develop diabetes early in life and, thus, have a particularly high lifetime risk of developing complications. In up to 20% of individuals with T1D, DKD leads to kidney failure requiring dialysis or kidney transplantation (8). Because of the improvements in the management and treatment of both diabetes and its complications (9), the 25-year cumulative incidence of DKD has halved in those diagnosed in the 1980s compared to those diagnosed in the 1970s. However, there was no further improvement in the later cohorts, and 36% of individuals with severe DKD still progressed to kidney failure within 15 years (6). DKD also substantially increases the risk of CVD, and as many as 40% of individuals with T1D and DKD develop CVD by the age of 40 (10).

DKD is characterized by urinary albumin excretion and gradually decreasing renal function, measured or estimated as glomerular filtration rate (eGFR). Urinary albumin excretion can be classified as normal or mildly increased, moderately, or severely increased albuminuria; the two latter ones are also called micro- and macroalbuminuria. The classical view has been that albuminuria represents an earlier sign of DKD, followed by reduced eGFR and eventually kidney failure, but a substantial proportion of individuals with DKD may present with reduced kidney function even without albuminuria (11). On the tissue level, DKD is characterized by glomerular and tubular basement membrane thickening, mesangial expansion, glomerulosclerosis, podocyte effacement, and, ultimately, nephron loss (12). It is of note, however, that kidney biopsies are rarely taken for diagnostic purposes. Therefore, any chronic kidney disease (CKD) in an individual with diabetes is *a priori* considered as DKD, irrespective of the underlying pathophysiology (11). Lack of a biopsy proof is less of a problem in T1D because most of the individuals with T1D and DKD have histologically true diabetic nephropathy.

DKD is a complex multifactorial disease in which both genetic and environmental risk factors contribute to the development and progression of the disease. However, the exact molecular mechanisms leading to DKD remain poorly understood. Apart from albuminuria and eGFR, no other biomarkers are yet in clinical use for monitoring disease progression or identification of individuals at risk, and only a few treatment options exist for the prevention of DKD, especially in individuals with T1D. To address these issues, genetic studies aim to identify the underlying molecular mechanisms leading to DKD. Here, we review the genetic factors that have been identified for DKD, mainly based on genome-wide association studies (GWASs) performed within the latest decade and summarize the main findings from epigenetic studies—being the potential dynamic link between genes and the environment—investigating the DNA methylation changes associated with DKD.

## 2 Heritability of DKD

Three decades ago, family studies reported clustering of DKD in siblings with T1D, suggesting an inherited component of the disease (13–17). More recently, a genome-wide estimation of the narrow-sense DKD heritability—the proportion of phenotypic variance explained by additive genetic factors—based on unrelated individuals with T1D reported 24%–42% heritability of DKD, depending on the phenotype definition. The heritability estimates were as high as 59% when adjusted for sex, diabetes duration and age at diabetes diagnosis, and with a tendency to higher heritability estimates for the more severe definitions (18). Similar analyses in individuals with T2D suggested only 8%–25% heritability for DKD, potentially reflecting more heterogeneous mechanisms leading to DKD in T2D in addition to a more important contribution of environmental factors (19, 20). Indeed, a sub-analysis of individuals with T2D from the Action to Control Cardiovascular Risk in Diabetes trial suggested that the gene–treatment interaction explains a large part of the phenotypic variance in microalbuminuria. Nevertheless, the heritability estimates for albuminuria and eGFR both in T1D and T2D range between 7% and 75% (19, 21–25).

## 3 Common genetic variants associated with DKD

### 3.1 Early genetic studies for DKD

The early genetic studies on DKD utilized various microsatellite markers and single-nucleotide polymorphisms (SNPs) for family-based linkage studies to identify chromosomal regions co-segregating with DKD. One of the strongest linkage peaks with a logarithm of odds (LOD) score of 3.1 was obtained in a candidate gene study of the *AGTR1* on chromosome 3q (26), and many genome-wide linkage scans reported a suggestive linkage peak on the extended 3q21-q29 region (27–31). Subsequent fine-mapping efforts of candidate genes on the 3q region, comparing the allele frequencies of tens or hundreds of SNPs in unrelated DKD cases and controls, suggested, e.g., *ADIPOQ* (32) and *NCK1* (33) to be involved in DKD. A linkage analysis in Turkish families with T2D and DKD identified a strong linkage peak on chr18q22.3–23 (LOD score = 6.1) (34), subsequently fine-mapped to a polymorphism in the *CNDP1* gene associated with both DKD and serum carnosinase concentrations (35).

In addition to the positional candidates, biological candidate gene studies were performed on the basis of information and hypotheses of the underlying biology. However, the results were mostly inconclusive, with limited statistical evidence due to the small sample number, lenient statistical threshold, and lack of external replication (36). The findings with the strongest statistical evidence include variants on the promoter region of the *EPO* gene encoding for erythropoietin [rs1617640, *p*-value = 2.7 × 10^−11^ (37)], as well as in the *SLC19A3* gene encoding for a high-affinity thiamine (vitamin B) transporter [rs12694743, *p* = 2.30 × 10^−8^ (38)], both associated with a combined phenotype of kidney failure and diabetic retinopathy.

### 3.2 Genome-wide association studies on DKD

To overcome the limitations of the candidate gene studies, the first GWASs covering hundreds of thousands of SNPs were pursued nearly two decades ago, identifying genetic risk factors for both T2D (39–41) and T1D (42). The GWASs have since identified thousands of genetic loci affecting common complex diseases, supporting the multifactorial genetic background and the common disease/common variant (CDCV) hypothesis that suggests that common genetic factors significantly contribute to the risk of common diseases and traits (43). Because of the burden of multiple testing of hundreds of thousands, or even millions of genetic variants, only associations reaching the stringent threshold of a *p*-value < 5 × 10^−8^ are considered genome-wide significant. The GWASs on DKD have to date identified 41 loci genome-wide significantly associated with various case-control definitions of DKD, as detailed in **Table 1**.

**Table 1:** Variants genome-wide significantly (*p*-value < 5 × 10^−8^) associated with DKD.

SNP	Reported gene	Diabetes population	Phenotype	N cases vs. controls	*P*-value	EA	NEA	OR	Refs
rs7583877	*AFF3*	T1D	ESKD	1,786 vs. 8,718	1.2 × 10^−8^	C	T	1.29	(44)
rs12437854	*RGMA/MCTP2*	T1D	ESKD	1,786 vs. 8,718	2.0 × 10^−9^	G	T	1.8	(44)
rs4972593	*SP3/CDCA7*	Women with T1D	ESKD	688 vs. 2,009	3.9 × 10^−8^	A	T	1.81	(45)
rs12523822	*SCAF8/CNKSR3*	T1D + T2D ^a^	DKD	5,226 vs. 8,510	1.3 × 10^−8^	G	C	0.73	(46)
rs56094641	*FTO*	T2D	DKD	4,022 vs. 6,980	7.7 × 10^−10^	G	A	1.23	(47)
rs9942471	*GABRR1*	T2D	Microalbuminuria	1,989 vs. 2,238	4.5 × 10^−8^	A	C	1.25	(19)
rs72858591	*RND3/RBM43*	T2D cases vs. non-diabetic controls	ESKD	3,432 vs. 6,977	4.5 × 10^−8^	C	T	1.42	(48)
rs58627064	*SLITRK3*	T2D cases vs. non-diabetic controls	ESKD	3,432 vs. 6,977	6.8 × 10^−10^	T	G	1.62	(48)
rs142563193	*ENPP7*	T2D cases vs. non-diabetic controls	ESKD	3,432 vs. 6,977	1.2 × 10^−8^	A	G	0.74	(48)
rs142671759	*ENPP7*	T2D cases vs. non-diabetic controls	ESKD	3,432 vs. 6,977	5.5 × 10^−9^	C	T	2.26	(48)
rs4807299	*GNG7*	T2D cases vs. non-diabetic controls	ESKD	3,432 vs. 6,977	3.2 × 10^−8^	A	C	1.67	(48)
rs9622363	*APOL1*	T2D cases vs. non-diabetic controls	ESKD	3,432 vs. 6,977	1.4 × 10^−10^	A	G	0.77	(48)
rs75029938	*GRAMD3*	T2D excluding APOL1 carriers ^b^	ESKD	2,768 vs. 6,059	2.0 × 10^−9^	T	C	1.89	(48)
rs17577888	*MGAT4C*	T2D excluding APOL1 carriers ^b^	ESKD	2,768 vs. 6,059	3.9 × 10^−8^	T	G	0.67	(48)
rs55703767	*COL4A3*	T1D	DKD	4,948 vs. 12,076	5.3 × 10^−12^	T	G	0.79	(49)
rs12615970	*COLEC11*	T1D	CKD	4,266 vs. 14,838	9.4 × 10^−9^	G	A	0.76	(49)
rs142823282	*TAMM41*	T1D	Microalbuminuria	2,477 vs. 12,113	1.1 × 10^−11^	G	A	6.75	(49)
rs145681168	*HAND2-AS1*	T1D	Microalbuminuria	2,477 vs. 12,113	5.4 × 10^−9^	G	A	5.53	(49)
rs118124843	*DDR1*	T1D	Microalbuminuria	2,477 vs. 12,113	3.4 × 10^−8^	T	C	3.78	(49)
rs77273076	*MBLAC1*	T1D	Microalbuminuria	2,477 vs. 12,113	1.0 × 10^−8^	T	C	9.12	(49)
rs551191707	*PRNCR1*	T1D	ESKD vs. macroalbuminuria	2,187 vs. 2,725	4.4 × 10^−8^	CA	C	1.7	(49)
rs144434404	*BMP7*	T1D	Microalbuminuria	2,477 vs. 12,113	4.7 × 10^−9^	T	C	6.75	(49)
rs115061173	*LINC01266*	T1D	ESKD	2,187 vs. 12,101	4.1 × 10^−8^	A	T	9.39	(49)
rs116216059	*STAC*	T1D	ESKD	2,187 vs. 17,216	1.4 × 10^−8^	A	C	8.76	(49)
rs191449639	*MUC7*	T1D	DKD	4,948 vs. 12,076	1.3 × 10^−8^	A	T	32.5	(49)
rs149641852	*SNCAIP*	T1D	CKD extreme	2,235 vs. 14,993	1.4 × 10^−8^	T	G	9.03	(49)
rs183937294	*PLEKHA7*	T1D	Microalbuminuria	2,477 vs. 12,113	1.7 × 10^−8^	G	T	17.3	(49)
rs61983410	*STXBP6*	T1D	Microalbuminuria	2,477 vs. 12,113	3.1 × 10^−8-^	T	C	0.79	(49)
rs113554206	*PAPLN*	T1D	Macroalbuminuria	2,751 vs. 12,124	8.5 × 10^−9^	A	G	4.62	(49)
rs185299109	*LINC00470/METTL4*	T1D	CKD	4,266 vs. 14,838	1.3 × 10^−8^	T	C	20.7	(49)
rs72763500	*NID1*	T2D	DKD	11,327 vs. 7,513	2.6 × 10^−8^	C	T	0.79	(50)
rs12917707	*UMOD*	T2D	DKD	11,327 vs. 7,513	4.5 × 10^−8^	T	G	0.86	(20, 50)
rs538044833^c^	*CCSER1*	T1D	CKD	727 vs. 3,962	2.8 × 10^−8^	C	T	3.0	(51)
rs72831309	*TENM2*	T1D + T2D	CKD + DKD	4,122 vs. 13,972	9.8 × 10^−9^	A	G	2.08	(52)
rs55703767	*COL4A3*	T1D + T2D	DKD	6,705 vs. 15,430	3.6 × 10^−11^	T	G	0.86	(52)
rs141560952	*DIS3L2*	Any diabetes vs. healthy controls	CKD	1,194 vs. 9,568	3.6 × 10^−9^	AGGG	A	192.6	(53)
rs425827	*KRT6B*	Any diabetes vs. healthy controls	CKD	1,194 vs. 9,568	2.7 × 10^−9^	A	T	5.31	(53)
rs73038008	*PLD1*	T1D or T2D	DKD^d^	1,973 vs. 5,734	1.7 × 10^−8^	C	T	2.55	(20)
rs77924615	*PDILT/UMOD*	T1D or T2D	DKD^d^	1,973 vs. 5,734	7.8 × 10^−9^	A	G	0.75	(20)
rs75733846	*WSCD2*	T2D	ESRD^e^	121 vs. 4,197	3.7 × 10^−8^	T	C	7.16	(20)
rs559427701	*SETDB2*	T2D	ESRD^e^	121 vs. 4,197	4.0 × 10^−9^	A	C	11.36	(20)
rs62202699	*LOC105372639*	T2D	Microalbuminuria^f^	702 vs. 2,210	4.3 × 10^−9^	T	C	2.97	(20)

SNP: Variant rs-identifier. EA: Effect allele. NEA: non-effect allele. OR: odds ratio. Refs: If multiple references are given, then the data in other columns for the same locus are taken from the first listed reference.

a Not all controls had diabetes.

b Controls did not have diabetes.

c Identified as underlying a linkage peak for DKD.

d CKD/DKD in self-reported, primary care, hospital, or death records.

e Dialysis or a rise of serum creatinine to 3.3 mg/dl (292 μmol/L).

f UACR ≥3.4 mg/mmol.

#### 3.2.1 Genome-wide association studies on DKD in type 1 diabetes

One of the first GWASs on DKD included 1,705 individuals with T1D from the Genetics of Kidneys in Diabetes (GoKinD) collection and suggested multiple putative susceptibility loci, including a variant in the *FRMD3* gene suggestively associated with DKD (*p*-value = 5.0 × 10^−7^) (54) and replicated by some of the subsequent studies (54, 55). Re-analysis of the data, including imputed variants, suggested additional loci, including *SORBS1* (56); variants in the same gene were also supported by a later GWAS including 1,462 additional individuals with T1D, but the association was attenuated in the replication (57).

The first GWAS meta-analysis on DKD combining data across multiple studies was undertaken by the Genetics of Nephropathy, an International Effort consortium. The GWAS meta-analysis discovery stage included 6,691 participants of European ancestry and with T1D from the GoKinD US, the Finnish Diabetic Nephropathy (FinnDiane) Study, and from the All Ireland-Warren 3-Genetics of Kidneys in Diabetes UK and Republic of Ireland (UK-ROI) Collection. The combined meta-analysis with 11,847 participants with T1D resulted in two loci, an intronic variant rs7583877 in *AFF3*, and an intergenic rs12437854 between in the *RGMA* and *MCTP2* genes associated with kidney failure in T1D with a *p*-value < 5 × 10^−8^. Furthermore, the authors reported a suggestive association for rs7588550 in the *ERBB4* gene associated with DKD (*p*-value = 2.1 × 10^−7^). *In vitro* analyses on a renal epithelial cell line suggested that *AFF3* influences the transforming growth factor–β1 (TGF-β1)–induced fibrotic responses (44).

Of note, nearly 90% of the GWAS findings are located on non-coding regions and are enriched for gene regulatory regions, rather than changing the protein amino acid sequence and structure (58, 59). The associated genetic variant does not necessarily affect the gene expression of the underlying or the closest gene, and, thus, a common challenge in GWAS is to identify the target gene of the non-coding regulatory variants. With large expression quantitative trait locus (eQTL) databases that are now available, one can link the genotypes to gene expression levels. On the basis of eQTL data from whole blood in the eQTLGen.org database, the rs7583877 variant in the *AFF3* gene is indeed associated with *AFF3* gene expression (*p*-value = 2.9 × 10^−19^) (60).

In the same consortium, an analysis stratified by gender identified a variant between the *SP3* and *CDCA7* genes associated with kidney failure in women (rs4972593, *p*-value = 3.9 × 10^−8^) (45). Multiple estrogen-responsive elements were predicted near rs4972593, and the *SP3* gene showed higher expression in kidney glomeruli in women (45). Furthermore, the Sp3 transcription factor directly interacts with the estrogen receptor-α (61) and regulates kidney-related genes such as *TGFBI*, *CD2AP*, and *VEGFA*, supporting its role in kidney failure in women with T1D.

The largest GWAS on DKD in T1D to date was performed by the Diabetic Nephropathy Collaborative Research Initiative (DNCRI) consortium, including up to 19,406 individuals with T1D and of European ancestry from 17 cohorts. The analysis comprised 10 different case-control definitions for DKD, based on either albuminuria, eGFR, or both. Altogether, 16 loci reached a *p*-value < 5 × 10^−8^, with the strongest association for a common missense mutation rs55703767 (Asp326Tyr) in the collagen type IV alpha 3 chain (*COL4A3*) gene, associated with a 21% lower risk of DKD (*p*-value = 5.3 × 10^−12^) (49). The gene encodes a major structural component of the glomerular basement membrane (GBM). In kidney biopsies of the Renin Angiotensin System Study (RASS) study participants with T1D and normal AER, the carriers of the protective variant had thinner GBM (49). The variant effect was dependent on glycemia, as the association at rs55703767 was observed only among individuals with HbA_1c_ ≥ 7.5% in the HbA_1c_-stratified sub-analysis of 4,321 FinnDiane participants with longitudinal HbA_1c_ measurements. Similarly, in the Diabetes Control and Complications Trial (DCCT), followed by the Epidemiology of Diabetes Interventions and Complications (DCCT-EDIC) study, the rs55703767 effect on DKD was stronger among those recruited in the secondary cohort and randomized to conventional treatment and therefore had higher HbA_1c_. Thus, the *COL4A3* rs55703767 association with DKD seems specific to diabetes and amplified by poor glucose control (49). The lead loci in the DNCRI meta-analysis also included other collagen-related findings: association with microalbuminuria for the rs116772905 variant in the *DDR1* gene encoding the epithelial discoidin domain-containing receptor 1, which binds collagens including type IV collagen; and gene aggregate analysis found variants in the *COL20A1* gene associated with severe CKD.

#### 3.2.2 Genome-wide association studies on DKD in type 2 diabetes

One of the first GWASs on DKD among individuals with T2D and the first transethnic meta-analysis of DKD included 4,909 individuals with T2D from the Family Investigation of Nephropathy and Diabetes (FIND) consortium in the discovery cohort and, altogether, 13,736 individuals in the final meta-analysis (including 6,229 non-diabetic controls). The analysis identified rs12523822 near the *SCAF8* and *CNKSR3* genes associated with a 43% lower risk of DKD in American Indians (*p*-value = 5.7 × 10^−9^) and with directionally consistent results across the ethnic groups (46). *CNKSR3* is a direct mineralocorticoid receptor target gene highly expressed in the renal cortical collecting ducts. The gene is involved in the transepithelial sodium transport and is upregulated in response to physiologic aldosterone concentrations (62). Clinically, renin-angiotensin-aldosterone system blockade is the main therapy for individuals with DKD and many other kidney diseases (63, 64). It is of note that the Finerenone in Reducing Kidney Failure and Disease Progression in Diabetic Kidney Disease (FIDELIO-DKD) trial with the non-steroidal mineralocorticoid-receptor-antagonist finerenone on top of standard of care showed cardio- and renoprotection in albuminuric individuals with T2D (65).

As end-stage kidney disease (ESKD) is disproportionately affecting African Americans (AAs), a subsequent FIND study GWAS focused on AAs and was extended to 3,432 T2D-ESKD cases and 6,977 non-diabetic non-nephropathy controls (*N* = 10,409), followed by a discrimination analysis in 2,756 T2D non-nephropathy controls to exclude T2D-associated variants. Six independent variants located in or near *RND3/RBM43*, *SLITRK3*, *ENPP7*, *GNG7*, *EFNB2*, and *APOL1* were associated with T2D-ESKD (*p*-value < 5 × 10^−8^), whereby variants in *EFNB2*, *GNG7*, and *APOL1* were also associated with all-cause ESKD (48). *EFNB2* encodes Ephrin-B2 and is expressed in the developing nephron and contributes to the glomerular microvascular assembly (66). The *APOL1* missense mutations rs73885319 (Ser342Gly), rs60910145 (Ile384Met), and rs71785313 (Asn388 and Tyr389 deletion), also known as the *APOL1* G1 and G2 haplotypes, are only found in individuals with African ancestry and are a major contributor to non-diabetic ESKD in AAs (48, 67, 68). To enrich for T2D-associated ESKD, an analysis excluding the *APOL1* ESKD-risk allele carriers identified additional variants in the *GRAMD3* (rs75029938, *p*-value = 2.0 × 10^–9^) and *MGAT4C* (rs17577888, *p*-value = 3.9 × 10^−8^) genes (48).

A GWAS in 7,614 Japanese individuals with T2D found the rs56094641 in the *FTO* gene to be associated with DKD (*p*-value = 7.6 × 10^−10^) (47). *FTO* is one of the strongest genetic loci for obesity and adiposity (69), and rs56094641 is in linkage disequilibrium (LD) with the obesity signal such that the DKD risk-associated allele is also associated with obesity. Indeed, other Mendelian randomization studies utilizing genetic information suggest that obesity is a causal risk factor for DKD (52, 70). However, the association between rs56094641 and DKD was not affected by adjustment for body mass index (BMI), suggesting that the locus affects DKD through another mechanism than an increase in BMI (47). Indeed, the *FTO* locus has been highlighted as a pleiotropic one, associated with multiple biomarkers and traits such as sweet vs. salty taste preference through modifying the regulatory properties of enhancers targeting the *IRX3* and *IRX5* gene expression in various tissues (71, 72).

The SUrrogate markers for Micro- and Macrovascular hard endpoints for Innovative diabetes Tools (SUMMIT) Consortium GWAS meta-analysis of DKD in T2D included 5,717 individuals of European ancestry and with T2D at the discovery stage. After joint analysis with additional European individuals, rs9942471 upstream *GABRR1*, encoding the rho1 subunit of the GABA type a receptor, was associated with microalbuminuria (*p*-value = 4.5 × 10^−8^), although the association did not replicate in Asian individuals or in individuals with T1D (19). The variant is in LD with the lead eQTL association signal for *GABRR1* expression in multiple tissues (19). Extended to individuals with T1D and other ethnicities, the joint meta-analysis involved up to 40,340 subjects with diabetes. However, meta-analysis with individuals with T1D (18) revealed no loci for dichotomous DKD phenotypes. Nevertheless, variants in the *UMOD* and *PRKAG2* loci, previously associated with eGFR and CKD in the general population (73, 74), were associated with eGFR also in individuals with diabetes (**Table 2**) (19).

**Table 2:** Variants associated with eGFR in diabetes.

SNP	Reported gene	Diabetes population	Phenotype	N total	*P*-value	EA	NEA	Beta	Refs
rs12917707 ^a,b^	*UMOD*	T1D + T2D	log eGFR per allele	11,522	2.5 × 10^−8^	T	G	0.0266	(19, 50, 75)
rs11864909 ^a^	*UMOD*	T1D + T2D	ml/min/1.73 m^2^	23,708	2.3 × 10^−12^	T	C	2.11	(19)
rs1974990	*SSB*	T1D + T2D	ml/min/1.73 m^2^	13,158	4.8 × 10^−8^	G	T	4.07	(19)
rs10224002 ^a^	*PRKAG2*	T1D + T2D	ml/min/1.73 m^2^	22,165	2.7 × 10^−8^	A	G	2.01	(19, 50)
rs267738 ^a^	*CERS2*	Any	log eGFR per allele	176,573	2.7 × 10^−8^	T	G	−0.0065	(76)
rs4665972 ^a^	*SNX17*	Any	log eGFR per allele	170,721	3.3 × 10^−9^	T	C	0.0057	(76)
rs10206899 ^a^	*ALMS1P*	Any	log eGFR per allele	143,419	1.6 × 10^−8^	T	C	−0.0068	(76)
rs1047891 ^a^	*CPS1*	Any	log eGFR per allele	170,741	5.6 × 10^−12^	A	C	−0.007	(76)
rs4663171	*SH3BP4*	Any	log eGFR per allele	170,901	8.8 × 10^−9^	A	T	−0.0072	(76)
rs28817415 ^a^	*SHROOM3*	Any	log eGFR per allele	176,910	9.9 × 10^−26^	T	C	−0.0091	(76)
rs10857147 ^a^	*FGF5*	Any	log eGFR per allele	170,848	2.4 × 10^−10^	A	T	−0.0061	(76)
rs434215 ^a,b^	*TPPP*	Any	log eGFR per allele	119,397	3.5 × 10^−19^	A	G	−0.0119	(76)
rs3812036 ^a^	*SLC34A1*	Any	log eGFR per allele	170,458	2.1 × 10^−12^	T	C	−0.0073	(76)
rs34246779 ^a^	*HMGN4*	Any	log eGFR per allele	172,626	1.1 × 10^−8^	A	G	−0.0091	(76)
rs3101824 ^a,b^	*SLC22A2*	Any	log eGFR per allele	176,569	3.6 × 10^−23^	T	C	−0.0143	(76)
rs11761603 ^a^	*UNCX*	Any	log eGFR per allele	168,668	4.8 × 10^−15^	T	C	0.0075	(76)
rs6464165 ^a^	*PRKAG2*	Any	log eGFR per allele	136,252	4.0 × 10^−21^	T	C	0.0107	(76)
rs9314272 ^a^	*STC1*	Any	log eGFR per allele	177,021	9.4 × 10^−10^	A	G	−0.0054	(76)
rs7033278 ^a^	*PIP5K1B ^c^*	Any	log eGFR per allele	176,480	1.3 × 10^−10^	T	C	0.0062	(76)
rs80282103 ^a^	*LARP4B*	Any	log eGFR per allele	176,591	6.8 × 10^−11^	A	T	0.0109	(76)
rs55917128	*LOXL4*	Any	log eGFR per allele	176,998	4.6 × 10^−8^	T	C	−0.0048	(76)
rs963837 ^a,b^	*DCDC5*	Any	log eGFR per allele	170,722	2.4 × 10^−34^	T	C	−0.0108	(76)
rs2004649 ^a^	*MAP3K11*	Any	log eGFR per allele	176,918	6.1 × 10^−10^	A	G	−0.0055	(76)
rs10899482 ^a^	*GAB2*	Any	log eGFR per allele	177,039	1.2 × 10^−8^	A	C	−0.0058	(76)
rs2461700 ^a^	*GATM*	Any	log eGFR per allele	177,144	1.5 × 10^−15^	T	C	0.008	(76)
rs17631603 ^a^	*WDR72*	Any	log eGFR per allele	177,042	6.6 × 10^−15^	A	G	0.0068	(76)
rs11636251 ^a^	*NRG4*	Any	log eGFR per allele	171,081	1.9 × 10^−14^	T	C	−0.0069	(76)
rs77924615 ^a,b^	*UMOD/PDILT*	Any	log eGFR per allele	170,741	1.9 × 10^-106^	A	G	0.0234	(76)
rs9895661 ^a^	*BCAS3*	Any	log eGFR per allele	176,461	6.7 × 10^−10^	T	C	0.0066	(76)
rs8096658 ^a^	*NFATC1*	Any	log eGFR per allele	167,173	1.6 × 10^−12^	C	G	0.0067	(76)
rs6015028 ^a^	*PCK1*	Any	log eGFR per allele	176,558	1.4 × 10^−9^	A	T	−0.0071	(76)
rs1882961 ^a,b^	*NRIP1*	Any	log eGFR per allele	176,630	3.6 × 10^−14^	T	C	−0.0073	(76)
rs9607518 ^a^	*MAFF*	Any	log eGFR per allele	170,649	2.2 × 10^−8^	T	C	−0.0049	(76)

SNP: Variant rs-identifier. EA: Effect allele. NEA: non-effect allele. Beta: effect size beta estimate. Refs: If multiple references are given, then the data in other columns for the same locus are taken from the first listed reference.

a Associated with eGFR also in the general population.

b Significant effect size difference between individuals with and without diabetes.

c DNA methylation of CpGs in the gene region associated with DKD (77, 78).

#### 3.2.3 Genome-wide association studies on DKD in combined diabetes populations

Meta-analysis of the DNCRI [T1D (49)] and SUMMIT consortia [both T1D (18) and T2D (19)], excluding the overlap between the consortia, and harmonized for the 10 phenotype definitions of DKD for available cohorts, included nearly 27,000 individuals with diabetes (52). The meta-analysis identified a novel intronic variant, rs72831309 in the *TENM2* gene, to be associated with a lower risk of the combined CKD-DKD phenotype (*p*-value = 9.8 × 10^−9^). *TENM2* gene expression in kidney tubules correlated positively with eGFR (*p*-value = 1.6 × 10^−8^) and negatively with tubulointerstitial fibrosis (*p*-value = 2.0 × 10^−9^). In addition, the gene-level analysis identified 10 genes significantly associated with DKD (*COL20A1*, *DCLK1*, *EIF4E*, *PTPRN–RESP18*, *GPR158*, *INIP–SNX30*, *LSM14A*, and *MFF*; *p*-value <2.7 × 10^−6^). Transcriptome-wide association study integrating GWAS with human glomerular and tubular gene expression data demonstrated a higher tubular *AKIRIN2* gene expression associated with DKD (*p*-value = 1.1 × 10^−6^). Expression of multiple lead genes correlated with renal phenotypes, e.g., tubular *DCLK1* expression correlated with fibrosis (*p*-value = 7.4 × 10^−16^) and *SNX30* expression with eGFR (*p*-value = 5.8 × 10^−14^), and negatively with fibrosis (*p*-value < 2.0 × 10^−16^) (52).

In addition to the disease-specific cohorts, large population-based biobanks allow analyses of an increasing number of samples and phenotypes. A GWAS on DKD in the UK Biobank included 13,123 unrelated individuals with diabetes and of European origin. Of note, the heritability estimate for DKD, defined based on ICD-10 codes (E11.2, T2D with kidney complications, or any CKD code assigned after diabetes) or a measurement of albuminuria or eGFR, was only 0.027 with a standard deviation (SD) of 0.03; heritability estimate for eGFR in T2D was higher, 0.1 with an SD of 0.01. GWAS on DKD and eGFR identified variants in the *UMOD* and *PRKAG2* loci (50). Meta-analysis with the SUMMIT T2D study further identified a novel variant, rs72763500, associated with the combined DKD definition. The variant is associated with alternative gene splicing of the *NID1* gene (50), encoding for nidogen-1, a sulfated glycoprotein involved in the development of GBM, where it binds to laminin and type IV collagen (79). Another study in the UK Biobank, although focused on heritability estimates for diabetic micro- and macrovascular complications, additionally found a variant rs73038008 near *PLD1* associated with DKD (self-reported or medical records); as well as variants in *WSCD2* and *SETDB2* associated with ESKD and in *LOC105372639* associated with microalbuminuria (20).

#### 3.2.4 Genome-wide association studies on albuminuria and eGFR in diabetes

**Figure 1:**
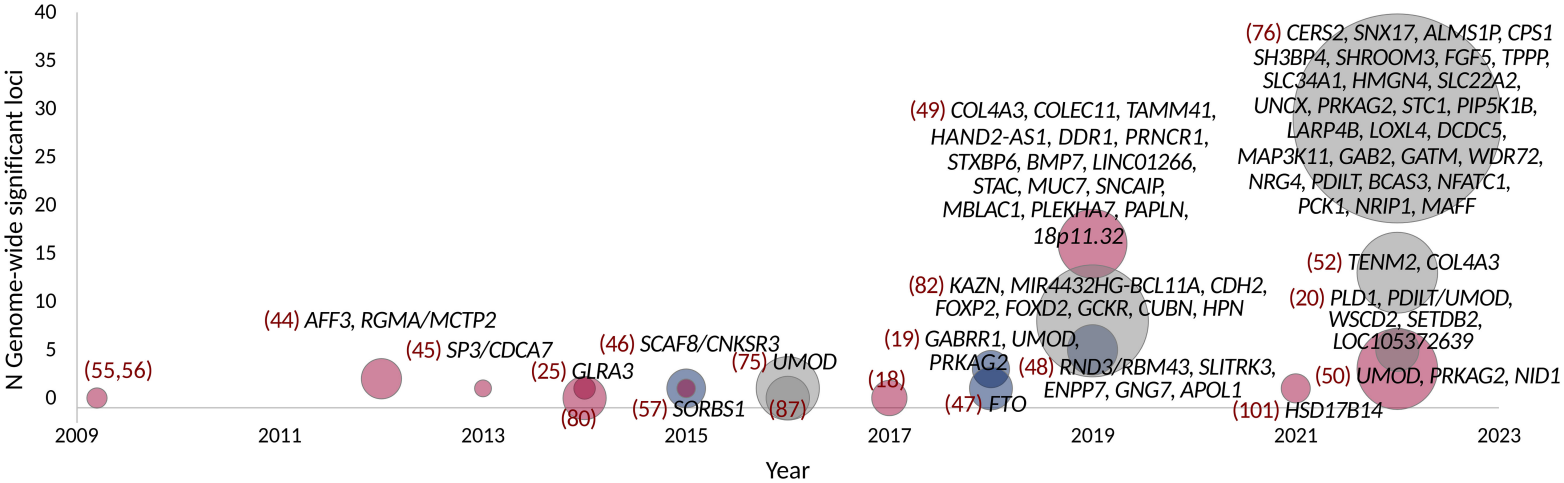
GWAS on DKD, albuminuria, and eGFR in diabetes. Point size indicates the number of samples. Studies with individuals with T1D are colored red, T2D with blue, and combined T1D + T2D, any type of diabetes or unspecified type of diabetes with gray. Gene names indicate loci reaching genome-wide significance (*p*-value < 5 × 10^−8^).

In addition to the dichotomous case-control definitions of DKD, GWASs have also explored albuminuria and eGFR as continuous traits in individuals with diabetes (**Figure 1**). Only few studies have identified variants with genome-wide significance for albuminuria (**Table 3**) or eGFR (**Table 2**), and most of these loci were identified in diabetes-specific sub-analyses of larger general population studies.

**Table 3:** Variants associated with albuminuria in diabetes.

SNP	Reported gene	Diabetes population	Phenotype	N	*P*-value	EA	NEA	Beta	Refs
rs10011025	*GLRA3*	T1D	log_10_ AER	1,925	1.5 × 10^−9^	G	A	0.21	(25, 81)
rs59825600	*KAZN*	Any	sd of log(UACR)	40,668	3.6 × 10^−8^	A	G	−0.075	(82)
rs6688849^a^	*FOXD2*	Any	sd of log(UACR)	51,215	4.1 × 10^−9^	A	G	−0.049	(82)
rs780093 ^a^	*GCKR*	Any	sd of log(UACR)	51,515	1.5 × 10^−13^	T	C	0.049	(82)
rs6706313	*MIR4432HG-BCL11A*	Any	sd of log(UACR)	51,162	2.8 × 10^−8^	A	G	−0.041	(82)
rs17137004	*FOXP2*	Any	sd of log(UACR)	51,294	2.7 × 10^−8^	A	G	−0.036	(82)
rs74375025 ^a^	*CUBN*	Any	sd of log(UACR)	50,641	1.1 × 10^−24^	A	G	0.106	(82)
rs4258701	*CDH2*	Any	sd of log(UACR)	51,328	1.1 × 10^−8^	T	C	0.039	(82)
rs149131600 ^a^	*HPN*	Any	sd of log(UACR)	46,939	3.5 × 10^−8^	T	C	0.050	(82)

SNP: Variant rs-identifier. EA: Effect allele. NEA: non-effect allele. Beta: effect size beta estimate. Refs: If multiple references are given, then the data in other columns for the same locus are taken from the first listed reference. AER, albumin excretion rate. UACR, urinary albumin-to-creatinine ratio.

a Significant also in the general population, but with larger effect in diabetes.

A GWAS including 1,925 Finnish individuals with T1D identified rs10011025 in the *GLRA3* associated with albuminuria (*p-*value = 1.5 × 10^−9^) (25). The association did not replicate in 3,771 other European individuals with T1D (*p*-value = 0.04, opposite direction) (25); however, the association was subsequently replicated in 1,259 additional Finnish individuals with T1D (81). The association was pronounced in individuals with HbA_1c_ > 7%. The *GLRA3* gene encodes the α3 subunit of glycine receptors. In pancreatic α-cells, glycine receptors stimulate glucagon release in response to glycine, thus counterbalancing the effects of insulin (83). Interestingly, the association with albuminuria was only evident among individuals with a 24-h urine collection. Because exercise can acutely increase albuminuria due to excess hemodynamic pressure (84), the authors hypothesized that the variant might affect renal sensitivity to hemodynamic pressure (81). Of note, in the eQTLGen database, the rs10011025 variant is associated with the expression of the *HPGD* gene, encoding for the 15-hydroxyprostaglandin dehydrogenase that catalyzes the prostaglandin catabolic pathway; prostaglandins are locally acting vasodilators and regulate renal hemodynamics in the kidneys (85).

Another GWAS on albuminuria included 54,450 individuals from the general population, confirming the previously identified *CUBN* locus (86) for albuminuria. In the sub-analysis of 5,825 individuals with diabetes, variants in the *HS6ST1* (rs13427836, *p*-value = 6.3 × 10^−7^) and *RAB38/CTSC* loci (rs649529, *p*-value = 5.8 × 10^−7^) were suggestively associated with albuminuria in subjects with, but not without diabetes (87). *RAB38* expression was found higher in the tubules of individuals with DKD compared to healthy controls, and *Rab38* knockout resulted in higher urinary albumin concentrations in diabetic rat models (87). A larger study including 564,257 individuals, of which 51,541 individuals with diabetes, identified eight loci associated with albuminuria in diabetes; all had larger effect among individuals with diabetes, and four (*KAZN*, *MIR4432HG*-*BCL11A*, *FOXP2*, and *CDH2*) were only found in the secondary analysis limited to diabetes (82).

Finally, a GWAS including 178,691 individuals with diabetes from the CKD Genetics (CKDGen) consortium and large biobank studies identified 29 genome-wide significant loci for eGFR, including 27 novel loci for eGFR in diabetes; among these, variants near *SH3BP4* and *LOXL4* were not associated with eGFR in the 1,296,113 individuals without diabetes (76).

### 3.3 Overlap between genetic factors for DKD and general population kidney traits

In the general population, nearly 900 genetic loci have been identified for eGFR in meta-analyses, including over 1.5 million individuals (88). Diabetes is one of the key risk factors for CKD, and 31% of the CKD-associated disability-adjusted life years can be attributed to diabetes (89). Other main risk factors for CKD include hypertension, obesity, and high age, all commonly seen among individuals with T2D in particular. In individuals with T1D, the majority of DKD is due to diabetic nephropathy. On the contrary, the renal lesions in kidney biopsies of DKD in T2D are heterogeneous, and a substantial proportion of the biopsies do not show the typical characteristics of diabetic nephropathy (90). However, kidney biopsies are rarely taken, and DKD is defined as any CKD in an individual with diabetes (91). Therefore, the question arises, how much of the genetic background of DKD is shared with the CKD and eGFR in the general population?

The DKD loci identified in individuals with T1D in the DNCRI consortium did not replicate in the general population GWAS for eGFR (49); conversely, the loci associated with eGFR in the general population (92) were not associated with DKD in T1D apart from the *UMOD* locus (49). On the contrary, some of the first findings for DKD in T2D included the *UMOD* and *PRKAG2* loci known from the general population (19), as well as the *APOL1* variant responsible for the majority of kidney failures in AAs (48). The CKDGen GWAS on eGFR including 133,413 individuals, of which 16,477 with diabetes, found that the effect size of the eGFR loci identified in the full population were highly correlated between individuals with and without diabetes (correlation coefficient of 0.80) (75). A more recent study on eGFR from the CKDGen consortium, including nearly 1.5 million participants of which 178,691 with T2D, systematically sought for differences in effect size between individuals with and without diabetes. They identified seven eGFR loci with significant difference in individuals with and without diabetes, as well as four loci with suggestive difference; in all but one, the effect was more pronounced or exclusively seen among individuals with diabetes (76). Similarly, in a GWAS for eGFR decline studied as a longitudinal trait in the general population, the effect sizes of the nine identified variants were on average two-fold higher in individuals with diabetes (93). Finally, the effect of the rs10795433 variant in the *CUBN* locus—the major locus for albuminuria—was larger among individuals with diabetes compared to those without diabetes (87). In addition, a rare *CUBN* variant rs141640975 had three times stronger effect in individuals with T2D compared with those without (94). Furthermore, rs141640975 was associated with higher eGFR but only in the non-diabetes population, suggesting pleiotropic effects on both kidney function measures (95).

In the DNCRI-SUMMIT GWAS meta-analysis for DKD, the similarity of DKD with kidney traits in the general population (of note, including individuals with diabetes) was assessed on a genome-wide scale instead of single-variant level, using the LD score regression approach. The albuminuria-based DKD definition, including microalbuminuria, was genetically correlated with microalbuminuria in the general population, both in the pooled analysis, and separately for individuals with T1D or T2D; of note, the correlation was over two-fold stronger in individuals with T2D. In addition, the eGFR-based CKD definition was also correlated with eGFR and CKD in individuals with T2D, but not in T1D despite more than three times more individuals with T1D (52). The analysis suggests that DKD in T2D has a larger proportion of shared genetic background with the general population, e.g., due to other co-existing risk factors such as aging, overweight, hypertension, and other glomerular diseases, while less overlap is observed between the general population kidney traits and DKD in T1D representing a purer form of diabetic nephropathy. The LD score regression with cardiometabolic and other traits further suggested that a proportion of the genetic background of DKD is shared with genetic risk factors, e.g., for aging (mother’s age at death), obesity, and smoking (52). However, the confidence intervals remain large, and further studies are needed to estimate the proportion of risk attributable to each risk factor.

Some interesting discrepancies also exist between DKD and the general population: For example, the missense variant rs55703767 in *COL4A3* is one of the strongest findings for DKD in T1D, but the effect is modified by glycemia, and the variant does not seem to affect kidney traits in the general population. On the contrary, variants in the flanking *COL4A4* (collagen type IV alpha 4 chain) gene were associated with albuminuria in the general population (rs57858280, *p*-value = 9 × 10^−11^) (82); according to the GTEx portal, the variant may affect the *COL4A4* splicing (https://gtexportal.org/ ). Rare mutations in both *COL4A3* and *COL4A4* cause Alport syndrome, a monogenic disease of basement membranes that frequently leads to ESKD, as well as thin basement membrane nephropathy and focal segmental glomerulosclerosis (96).

### 3.4 Overlap between genetic factors for DKD and diabetes

Some studies have suggested a correlation between the genetic risk factors predisposing to insulin resistance or T2D and DKD (18, 19, 52). Of note, these studies found no correlation between genetic risk factors predisposing to T1D and DKD. T2D was modestly causally associated with DKD in a Mendelian randomization study of individuals with either T1D or T2D (*p*-value = 0.02), but only obesity related traits remained significantly associated with DKD when using methods accounting for pleiotropic effects (52). However, among the lead variants for DKD, albuminuria, or eGFR in diabetes, only the albuminuria-associated *FTO* locus [rs56094641 (47)] has been associated with T2D. In addition, the albuminuria-associated rs780093 (82) in the highly polygenic *GCKR* locus, as well as the eGFR-associated rs4665972 (in *SNX17*, but in LD with variants mapped to *GCKR*), rs11864909 (*UMOD*), rs10206899 (*ALMS1P*), rs10899482 (*GAB2*), and rs9607518 (*MAFF*), are in LD with variants associated with T2D (https://ldlink.nci.nih.gov/?tab=ldtrait ; search for any “diabetes” in GWAS Catalog for variants in LD (*R* ^2^ ≥ 0.8 in European population), 21 March 2023), providing some evidence of genetic overlap between T2D and eGFR in diabetes.

## 4 From common to rare genetic variants for DKD

While common variants have a large effect on complex traits at the population level (43), the low frequency and rare variants can have a high impact on the individual level (97). In particular, protein-altering variants (PAVs), i.e., exon variants that change the protein amino acid sequence, can directly impact protein function. For example, 71% of severe *LDLR* mutation carriers had hypercholesterolemia in the UK Biobank WES data (98). To identify chromosomal regions harboring rare variants for DKD, a linkage study based on GWAS data of 6,019 FinnDiane study participants included 177 small pedigrees such as sib-ships, parent-offspring pairs, and more distant relations, with, altogether, 452 individuals, all with T1D. Eight chromosomal regions reached a significant LOD score > 3.3 (51). Many of these regions harbor genes in which mutations cause rare syndromes with kidney complications, such as *ARHGAP24* associated with focal segmental glomerulosclerosis (99) and *FRAS1* associated with the familial Fraser syndrome (100). Overlap with loci causing rare kidney syndromes supports the role of rare variants in the development of DKD. Interestingly, one suggestive linkage peak was observed in the *NID1* locus, recently associated with DKD in T2D (50). While a rare rs538044833 variant in the *CCSER1* locus was externally replicated (*p*-value = 2.8 × 10^−8^), the resolution remains low even in the GWAS-based linkage studies, hindering further fine-mapping and interpretation of the results.

In addition, on the basis of GWAS data, enriched for rare PAVs with the ExomeChip array, a gene aggregate meta-analysis including 4,196 individuals with T1D found PAVs in the hydroxysteroid 17-*β* dehydrogenase 14 (*HSD17B14*) gene exome-wide significantly (*p*-value < 5 × 10^−7^) associated with the disease progression from DKD to kidney failure. The gene and protein expression were attenuated in human diabetic proximal tubules and in mouse kidney injury models (101).

The GWAS genotyping chips cover only a portion of the PAVs, and genotype imputation quality largely depends on the variant minor allele count in the reference sample and can be limited for rare variants (102, 103). A whole-exome sequencing (WES) on DKD, including 997 individuals with T1D, did not find any variants or genes reaching robust exome-wide significance (18) but found suggestive evidence of association, e.g., for PAVs in the *THADA* gene, previously associated with T2D (104). A WES of 593 DKD cases and 2,066 healthy controls of European and African ancestry, with subsequent discriminatory analyses and replication in up to 11,487 multi-ancestry participants from the Trans-Omics for Precision Medicine study, identified an in-frame insertion rs141560952 in the *DIS3L2* gene (*p*-value = 3.6 × 10^−9^), and a *KRT6B* splice-site variant rs425827 associated with DKD (*p*-value = 2.7 × 10^−9^). Both variants were associated with DKD also when compared with diabetes controls without DKD, but with lower statistical significance (*p*-value = 1.4 × 10^−4^ and 2.8 × 10^−4^). Furthermore, gene aggregate analyses identified *ERAP2* (*p*-value = 4.03 × 10^−8^) and *NPEPPS* (*p*-value = 1.51 × 10^−7^); both are expressed in the kidney and implicated in the renin-angiotensin-aldosterone system–modulated immune response (53). However, the discriminatory analyses suggest that the *ERAP2* and *NPEPPS* may be primarily associated with diabetes *per se*, subsequently leading to DKD (53).

While WES mainly covers the protein-coding sequence, a whole-genome sequencing (WGS) study of 76 Finnish sibling pairs with T1D but discordant for DKD found significant enrichment of variants in DKD in gene promoter and enhancer regions, as well as for specific transcription factor binding sites (105), but larger studies are required to pinpoint the most relevant regulatory regions. Gene aggregate analysis of PAVs suggested protein kinase C isoforms (*PRKCE* and *PRKCI*) and protein tyrosine kinase 2 (*PTK2*) involved in DKD (105); of note, a recent GWAS on albuminuria in the general population highlighted variants in the *PRKCI* and demonstrated that a podocyte-specific deletion of aPKClambda/iota in mice results in severe proteinuria (82). A recent multi-ethnic WGS in 23,732 individuals identified three novel rare intronic variants for eGFR in the general population (106), and larger WGS for DKD are needed to identify the rare variants contributing to DKD.

## 5 Epigenetic factors for DKD

Studies focusing on epigenetic modifications have emerged in an increasing number during the last years. Epigenetic modifications can be described as chemical modifications of the DNA (or RNA) that can induce changes in gene expression without changing the underlying sequence. In contrast to an individual’s genetic variation, which is constant across tissues and throughout lifetime, epigenetic modifications are dynamic and modifiable. Thus, epigenetic changes may vary between tissues, cell types, and developmental stages and can even be affected by environmental factors. Furthermore, in disease states, the methylation patterns can change either as a cause or a consequence of the disease (107). In this way, epigenetic factors provide a link between the genome and the environment and can potentially reflect an individual’s risk of developing a disease more accurately at a given time. Although epigenetic changes are dynamic, there is evidence that epigenetic modifications, such as DNA methylation, persist in blood years after acute illness or metabolic changes in the body (108, 109). Consequently, epigenetic factors have been suggested as an underlying mechanism for metabolic memory (110, 111). Metabolic memory in diabetes refers to the sustained harmful effect of hyperglycaemia on diabetic complications, initially observed in the DCCT-EDIC study, even after improved glycaemic control (112, 113). In line with this observation, subsequent work in DCCT-EDIC has identified several epigenetic changes associated with metabolic memory (110, 111). A combination of DNA methylation levels at several HbA_1c_-associated sites explained as much as 71 to 97% of the association between HbA_1c_ and diabetic complications in the DCCT (114), further reinforcing the connection between epigenetic changes and metabolic memory.

DNA methylation is the most frequently studied epigenetic modification and occurs at cytosine bases of cytosine–phosphate–guanine dinucleotide sites (CpGs) in the DNA sequence. In addition to DNA methylation, additional epigenetic modifications exist, such as histone modifications (acetylation and methylation), and their role in DKD has also been explored. For example, dysregulation of histone H3 lysine 27 trimethylation (H3K27me3) in TGF-β1–induced gene expression has been associated with DKD (115). Histone modifications associated with DKD are reviewed, e.g., in (116), and are out of the scope of this review, where we focus on DNA methylation changes.

### 5.1 Various study settings for DNA methylation

Although whole-genome bisulfite sequencing for the analysis of the methylome has been done for DKD, sample sizes have been small (117). Studies assessing DNA methylation patterns across the genome, known as epigenome-wide association studies (EWASs) or methylome-wide association studies (MWASs), have primarily relied on Illumina’s BeadChip platforms, which have evolved from the Illumina 27K array with only ~27,000 sites to the Illumina 450K with ~450,000 and the EPIC array containing methylation levels at ~850,000 sites. However, this number of CpGs only accounts for a small amount of all the CpGs in the genome, totalling up to ~30 million (118). The EWASs have applied various significance thresholds, but a *p*-value below 9 × 10^−8^ has been suggested as a threshold for robust significance, adequately controlling for the false positive rate for the EPIC array (94). The genome-wide significance threshold recommended for Illumina’s 450K BeadChip is *p*-value < 2.4 × 10^−7^ or *p*-value < 3.6 × 10^−8^ (119), although the false discovery rate (FDR) has been widely used (**Table 4**). Contrary to the GWAS, which initially yielded few significant loci with increasing number of findings with larger studies, in EWAS, the use of varying thresholds, combined with unaddressed inflated test statistics especially in the early EWAS (131), has led to a quite varying number of identified methylation loci in the studies performed so far.

**Table 4:** EWASs on kidney disease and related traits in individuals with diabetes.

Study	Ethnicity	Tissue	Phenotype	Cases	Controls	N Total	CpGs (array)	p-threshold	N significant CpGs
Bell, 2010 (120)	White European	Blood	DKD	96 (T1D: 100%)	96 (T1D: 100%)	192	27,578 (27K)	*P_FDR_* < 0.05	19 (*P_FDR_* < 0.05); none with *P_FDR_ <*10^−8^
Sapienza, 2010 (121)	African American/Hispanic	Saliva	DKD	24 (T2D: 87%, T1D: 13%)	24 (T2D: 100%)	48	27,578 (27K)	Diffscore** > 20 or < −20	2,870, of which 30 remained significant after FDR adjustment (*P_FDR_* < 0.05)
Smyth, 2014 (122)	White European	Blood	CKD/DKD	255 (T1D: 44%)	152 (T1D: 74%)	407	485,577 (450K)	*P_FDR_* < 10^−8^	52 CpGs (*P_FDR_* < 10^−8^) in 23 genes
Swan, 2015 (123)	White European	Blood	DKD	196 (T1D: 100%)	246 (T1D: 100%)	442	450* (27k, 450K)	*P_FDR_* < 10^−8^	54 (*P_FDR_* < 10^−8^)
Qiu, 2018 (124)	American PIMA Indians	Blood	eGFR; ESKD; eGFR slope	80 (T2D: 100%)	101 (T2D: 100%)	181	397,063 (450K)	*P_FDR_* < 0.05	eGFR and ESKD: none (*P_FDR_* < 0.05); 77 (eGFR slope, *P_FDR_* < 0.05)
Gluck, 2019 (125)	Mixed	Kidney tubules	degree of kidney fibrosis	91 (22 with DKD)	0	91	321,473 (450 K)	*P_FDR_* < 0.05	Degree of fibrosis: 203 (*P_FDR_* < 0.05) of which 65 replicated (p < 0.05)
Sheng, 2020 (126)	Mixed	Blood	eGFR, eGFR slope, albuminuria	473 (all with diabetes)	0	473	866,836 (EPIC)	*P* < 5 × 10^−5^ (discovery), p < 6.4 × 10^−8^ (Bonferroni)	Albuminuria: 73 (*P* < 5 × 10^−5^), eGFR: 99 (*P* < 5 × 10^−5^); 1 (6.4 × 10^−8^), eGFR slope: 111 (*P* < 5 × 10^−5^); 3 (6.4 × 10^−8^)
Smyth, 2020 (127)	White European	Blood	DKD	150 (T1D: 100%)	100 (T1D: 100%)	677	482,421 (450K)	*P_FDR_* < 10^−8^, Δβ > 0.2	22
Kim, 2021 (128)	East Asian	Blood	DKD	87 (T2D: 100%)	80 (T2D: 100%)	167	749 315 (EPIC)	*P_FDR_* < 9.0 × 10^–8^	3 (*P_FDR_* < 9.0 × 10^–8^)
Smyth, 2021 (129)	White European	Blood	ESKD (4 analysis models)	107 (T1D: 100%)	253 (T1D: 100%)	360	862,927 (EPIC)	*P_FDR_* < 10^−8^, FC ± 2	36 (*P_FDR_* < 10^−8^, FC ± 2 across all four models)
Lecamwasam, 2021 (130)	Mixed	Blood	late CKD (eGFR<45) vs. early (eGFR≥45)	38 (T1D: 8%, T2D: 87%)	83 (T1D: 20%, T2D: 80%)	119	764 333 (EPIC)	*P_FDR_* < 0.05	1 (*P_FDR_* < 0.05)
Smyth, 2022 (77)	White European	blood	DKD (3 analysis models)	651 (T1D:100%)	653 (T1D: 100%)	1304	763 064 (EPIC)	*P_FDR_ <*9 × 10^−8^	32 (*P_FDR_* < 9 × 10^−8^)

27K, Illumina Infinium HumanMethylation 27K; 450K, Illumina Infinium HumanMethylation 450K; EPIC, Illumina Infinium HumanMethylation EPIC v1.

*Only CpGs within mitochondrial genes were surveyed.

**Diffscore = 10sgn(b-value_ESRD_ − b-value_diabetes no nephropathy_) log10p.

Most EWASs performed on DKD have examined DNA methylation in blood. Still, other tissues have been used, such as kidney samples micro-dissected into kidney tubules (125) and even saliva (121). The epigenetic changes observed in the kidney tissue likely reflect the local changes more accurately. Indeed, EWAS on fibrosis in kidney tissue samples identified 65 differentially methylated CpGs that were enriched on kidney regulatory regions (125). Another promising target tissue for studying kidney disease would be the urine, which can be collected non-invasively and easily from larger datasets. Urine, however, contains few nucleated cells and extracting a sufficient amount of DNA from urine has turned out to be a challenge (132).

### 5.2 Over 150 CpGs associated with DKD and related traits

**Figure 2:**
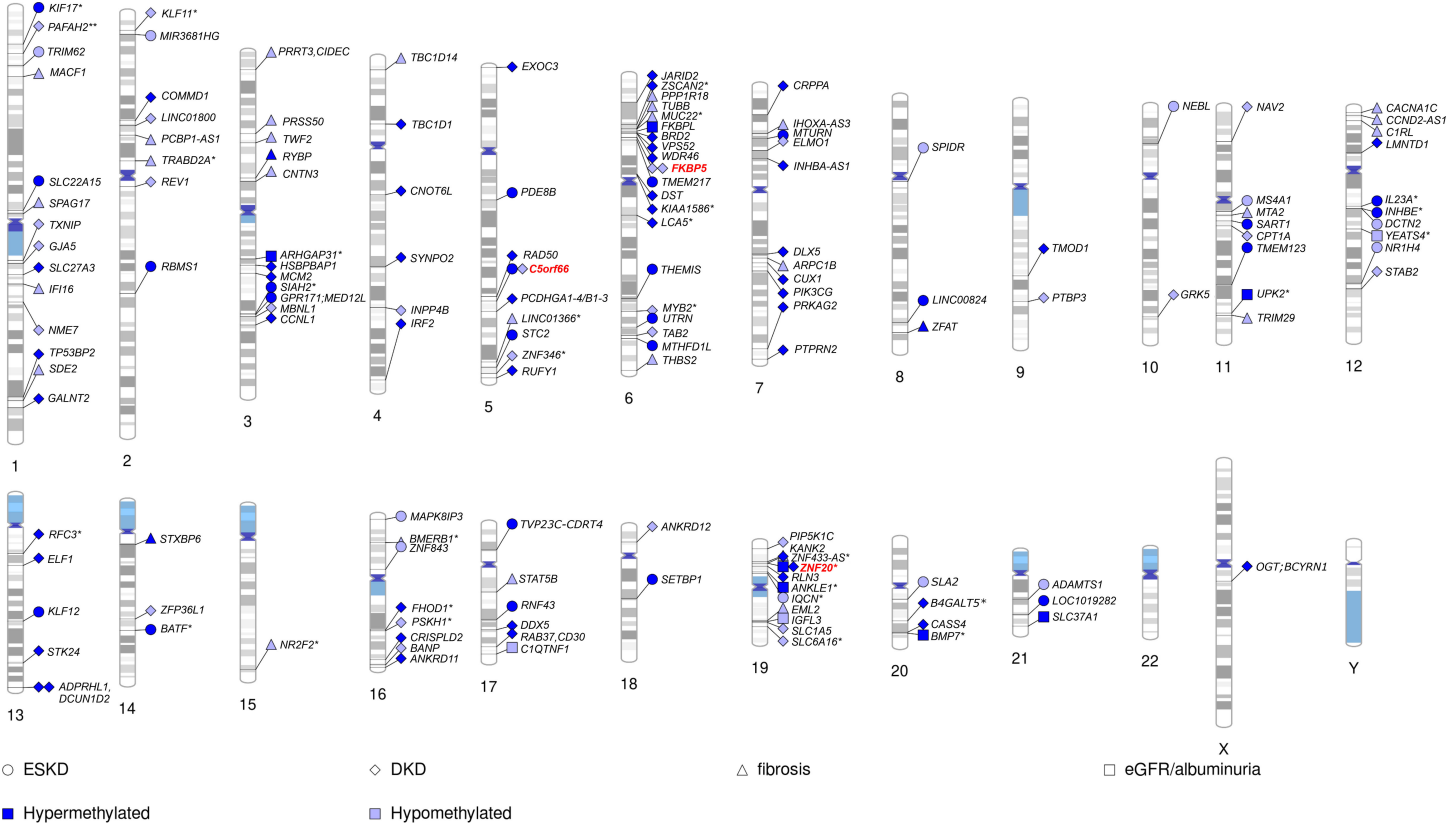
Chromosomal ideogram including CpGs methylation associated with kidney disease (DKD and ESKD), fibrosis, eGFR, or albuminuria in diabetes. For intergenic CpGs (*), the nearest gene is given. Hypermethylated CpGs (in kidney disease vs. controls) are denoted by a dark blue colour and hypomethylated by a light blue colour. CpGs appearing among top loci in multiple studies on kidney disease in diabetes denoted by a red color.

To date, methylation levels at over 150 CpG sites across the genome have been associated with DKD, eGFR, or albuminuria (*p*-value < 9 × 10^−8^), in studies including both T1D and T2D (**Figure 2**; **Table 4**; **Supplementary Table 1**), with the majority assessing DNA methylation in blood. The first DKD-EWAS identified DNA methylation levels at 19 CpGs associated with DKD in T1D (FDR < 0.05) using Illumina’s 27K array (120), highlighting one CpG located upstream of the *UNC13B* gene. An intronic SNP (rs2281999) in the same *UNC13B* gene was identified for DKD in T1D in a prior genetic association study including genetic variants in 127 candidate genes (133). More recent methylation arrays, with higher coverage have enabled identification of additional CpGs. Using the 450K array, Smyth et al. identified 53 CpGs within 23 genes with differential methylation in participants with CKD, of which approximately half had T1D. Of the 23 genes, six were in genes that are biological candidates for kidney disease: *CUX1*, *ELMO1*, *FKBP5*, *INHBA-AS1*, *PTPRN2*, and *PRKAG2* (122). Of these, genetic variants within the *PRKAG2*, encoding a protein kinase involved in cellular energy metabolism, have also been associated with eGFR in GWAS on kidney disease, both in individuals with and without diabetes (19, 73, 74). Following this study, several EWAS have been performed (**Table 4**), focusing mainly on DKD (77, 123, 127) and ESKD (129) in T1D but also on DKD in T2D (128) or eGFR in individuals with diabetes of unspecified/mixed type (124, 126, 130), yielding a plethora of sites that are differentially methylated, shown in **Figure 2** (CpGs with *p*-value < 9 × 10^−8^). The most recent and largest study, including 1,304 individuals with T1D, identified 32 sites with altered methylation in DKD (77), of which 23 were specific to the EPIC array. Methylation levels at seven CpGs were epigenome-wide significantly and differentially methylated after accounting for differences in multiple clinical risk factors (HbA_1c_, HDL cholesterol, triglycerides, BMI, smoking, and duration of diabetes), in addition to age, sex, and six cell-type proportions. These seven included two intergenic CpGs on chromosome 19 and four CpGs located within genes *PTBP3*, *NME7*, *SLC1A5*, and *SLC27A3* and one CpG within a long non-coding RNA (*LINC01800*).

Methylation levels at only a few CpGs have been associated with DKD in multiple studies (**Figure 2**). This can partly be explained by the higher coverage of Illumina’s EPIC array, with many CpGs on that array not present on previous arrays and, therefore, not testable. Consequently, one-third of the differentially methylated CpGs identified for DKD or eGFR in studies using the EPIC array (77, 126, 129) were novel and not available on previous arrays (**Supplementary Table 1**). However, methylation loci that have been repeatedly associated with DKD, include CpG within genes *C5orf66*, *FKBP5* (77, 122), and *PIP5K1C* (77, 129). In addition, higher methylation at the intergenic CpG cg17944885, located on chromosome 19 within a zinc finger gene cluster, has been repeatedly associated not only with DKD and eGFR in diabetes (77, 126, 130) but also with CKD and eGFR in the general population (78, 134, 135), as well as eGFR in other more specific cohorts, such as men with human immunodeficiency virus (HIV) (136). Moreover, CpGs within the *IRF2* (cg05165263) and *SLC27A3* (cg21961721) gene, both with higher methylation levels in DKD in T1D (77), have also been associated with eGFR (*p*-value = 5 × 10^−5^ and 8 × 10^−5^) in the general population (135), although not among the reported top loci.

Although most of the DNA methylation association studies performed on DKD have covered the whole genome, targeted approaches have been undertaken as well. Swan et al. evaluated DNA methylation levels associated with DKD for CpGs located within genes influencing mitochondrial function in 442 individuals with long term T1D (123). Although methylation levels at several CpG sites reached the threshold for epigenome-wide significance (*p*-value < 9 × 10^−8^), none of the differentially methylated CpG sites has emerged in subsequent EWASs.

A few CpGs identified as differentially methylated in DKD to date (**Figure 2**) also appear in EWAS on traits that are considered risk factors for DKD. Lower methylation of cg19693031 located in the 3′-untranslated region of the *TXNIP* gene has been recurrently observed in the context of diabetes and glycemia, such as persistently higher HbA_1c_ both in T2D and T1D (109, 126, 137). *TXNIP* encodes for the thioredoxin-interacting protein, which by binding to thioredoxin induces oxidative stress and apoptosis. Although it is mainly considered a glycemia-related methylation locus, it not only shows repeated associations with albuminuria and DKD (77, 109), explaining alone up to 45% of the HbA_1c_ association with DKD (114), but also associates with DKD and triglycerides independently of HbA_1c_ (109). Intriguingly, methylation levels at cg19693031 are also under genetic influence by SNPs located within the *SLC2A1* gene encoding for the glucose transporter 1 (GLUT1) (109). A recent EWAS on DKD performed a systematic trait enrichment analysis and found significant overlap with EWAS findings for traits and diseases such as aging, smoking, systolic and diastolic blood pressure, eGFR, and HbA_1c_ (77). Our lookup of the significant CpGs identified for DKD, eGFR, fibrosis, and albuminuria to date (**Figure 2**; 160 CpGs as listed in **Supplementary Table 1**) in the EWAS catalogue (associations with *p*-value < 9 × 10^−8^; http://www.ewascatalog.org , accessed 31 January, 2023) found an overlap with DKD risk factors including dyslipidemia (CpGs within *SLC1A5*, *TXNIP*, and *CPT1A*), HbA_1c_ (*TXNIP*), blood pressure (CpGs within *SLC1A5*, *TXNIP*, *CPT1A*, and *PTBP3*) and obesity (CpGs within *SLC1A5*, *TXNIP*, *CPT1A*, and *FKBP5*; **Supplementary Table 2**). For example, in the *CPT1A* gene, methylation at cg17058475 was associated with DKD in T1D (77) and has been robustly associated with the triglycerides (118) in the general population. *CPT1A* encodes a key enzyme in the fatty acid metabolism, namely, the hepatic isoform of carnitine palmitoyl transferase 1 (138), controlling the fatty acid flux in the liver. In addition, the genetic variants in the gene were also associated with triglycerides and HDL cholesterol in a recent GWAS (139).

### 5.3 DNA methylation for prediction of DKD

Several studies have provided evidence suggesting that changes in DNA methylation patterns could be used to predict DKD or its progression. Using 91 kidney tissue samples, Gluck et al. found that information from 471 differentially methylated CpGs in the kidneys helped them to predict kidney disease progression (125). However, the utility of kidney tissue–specific DNA methylation patterns as potential biomarkers remain limited, as individuals with DKD do not routinely undergo kidney biopsy. As an alternative, a study with methylation data from 831 individuals constructed methylation risk scores for 607 phenotypes based on electronic health records and suggested that blood methylation was particularly good in identifying individuals with pre-existing kidney failure and related traits (140). An EWAS in 181 American Indians with diabetes identified methylation levels at 77 CpG sites associated with eGFR decline over a 6-year period (124). Methylation at two CpGs (cg25799291 and cg22253401 in *FSTL5*) improved prediction of eGFR decline even when baseline eGFR and Albumin-to-creatinine ratio (ACR) were included in the model (124). In addition, in T1D, methylation levels at baseline can be used to predict progression of DKD. In total, 20 of the 32 differentially methylated CpGs in DKD in T1D predicted future progression to kidney failure in 397 individuals with DKD, 13 even after accounting for eight clinical risk factors (77). Furthermore, methylation at the two intergenic CpGs located within the zinc finger gene cluster on chromosome 19 predicted kidney failure, independent of baseline eGFR.

### 5.4 Epigenetic changes—the cause or the consequence?

Because of the dynamic nature of epigenetic changes, the methylation changes observed at CpGs in DKD can be either a cause or a consequence of the disease. To separate the causal methylation changes from the consequential, EWASs have also attempted Mendelian randomization, which uses genetic information to infer causality (77, 126, 128). Although these analyses have been partly hampered by the lack of genetic variants influencing CpG methylation, some causal associations have been observed. For example, Mendelian randomization suggested that higher methylation levels at cg23527387 located within the *REV1* gene reduces the risk of DKD in T1D (77). On the other hand, no evidence for causality was found for cg19693031 (*TXNIP*) or cg17944885 (between *ZNF788P* and *ZNF625-ZNF20*), suggesting that methylation changes observed at these sites are consequential to kidney disease or its other manifestations, e.g., hyperglycemia. Kim et al. used Mendelian randomization in the opposite direction, i.e., to assess the causal effect of metabolic phenotypes on CpG methylation changes identified in their EWAS on T2D (n = 8) and DKD (n = 3). These analyses revealed that fasting glucose resulted in 2% hypomethylation of cg00574958 located in the *CPT1A* gene, whereas HbA1c or BMI did not causally affect the cg00574958 methylation. Genetically determined eGFR, however, was associated with 7% hypomethylation of cg19693031 within *TXNIP* (*p*-value = 0.045), as well as hypomethylation of all the CpGs identified for DKD in T2D, including three CpGs within genes: *COMMD1*, *TMOD1*, and *FHOD1*.

## 6 Discussion

During the last 5 years, both GWAS and EWAS have identified an expanding number of genetic loci for DKD. Nearly 80 genetic loci have reached genome-wide statistical significance for DKD, albuminuria, or eGFR in diabetes to date. Much of this increase is not only due to larger meta-analyses of existing diabetes cohorts but also due to CKD studies in the general population including a substantial number of individuals with diabetes, as well as general population biobank studies. Even larger meta-analyses combining multiple biobank studies are likely to result in more genetic loci contributing to DKD. One of the major challenges of such studies will be how to best ascertain cases with DKD, either based on ICD codes that do not capture DKD well, self-reported DKD, or single measurements of albuminuria or eGFR, both of which vary over time. General population biobanks may also be affected by selection bias including healthier than average individuals (141), leading to a limited number of individuals with severe DKD or ESKD or with long-lasting diabetes: As DKD takes decades to develop (6), ideal study controls would only include individuals with diabetes without DKD despite a long diabetes duration.

The number of identified genetic loci now also allows comparison of the findings and the genetic overlap between general population CKD and DKD in T1D and T2D. The general population loci for eGFR seem to affect eGFR also in individuals with diabetes, especially those with T2D (76). For some variants, the effect size is markedly higher in the individuals with diabetes than in those without (e.g., UMOD, rs77924615, beta_DM_ = −0.019, beta_noDM_ = −0.011, *P*_diff_ = 1.3 × 10^−27^; *TPPP*, rs4663171, beta_DM_ = −0.011, beta_noDM_ = −0.004; *P*_diff_ = 2.5 × 10^−9^), potentially reflecting the elevated risk and accumulated risk factors for kidney complications among individuals with diabetes. On the other hand, genetic risk factors for DKD in T1D seem to differ from the general population (52). These support the notions from the clinical and epidemiological studies suggesting that individuals with T2D can have either DKD, non-DKD, or both, whereby individuals with T1D mainly develop diabetic nephropathy with a different pathophysiology from the general CKD (11, 90). Therefore, future genetic studies on DKD will need to balance between maximizing the number of samples (any diabetes, or even the general population with focus on diabetes) but with a more heterogeneous phenotype, and a cleaner DKD phenotype in T1D with diabetic nephropathy as a more likely underlying cause, but with a more limited number of samples.

GWASs on DKD have been performed in various populations beyond the European ancestry (46–48), and some of the identified variants are population-specific, e.g., the *APOL1* variants associated with all-cause and diabetic ESKD in AAs (48, 67, 68). For many complex diseases, such as T2D, extension to further populations, as well as larger multi-ancestry GWAS meta-analyses have yielded novel genetic susceptibility loci by increasing the total sample size and capturing additional variants with ancestry-correlated heterogeneity in the allelic effect sizes (104, 142). Multi-ancestry GWASs also provide improved fine-mapping resolution of the detected association signals, i.e., can provide a smaller number of variants in the credible set including the underlying causal variant among the many associated ones (142). Therefore, such multi-ancestry studies are likely to reveal novel loci with improved fine-mapping for DKD as well. On the contrary, homogenous study populations may be particularly important in sequencing studies aiming to identify rare genetic risk factors for DKD.

Although there are known differences in the methylation pattern of a number of CpGs between different ethnicities (143), there is a lack of ethnic diversity in EWAS, which are based mainly on individuals of European ancestry (144, 145). A recent multi-ancestry EWAS on kidney function (135) revealed several population-specific methylation patterns for eGFR in the general population with little overlap between African and European populations. These discrepancies, however, could be due to both genetic and environmental differences between the different ethnic groups. The expansion of EWAS datasets in DKD to include multi-ancestry populations is still lacking.

The GWASs have also enabled creation of polygenic risk scores (PRSs) that may be used for risk stratification and identification of affected traits and phenotypes. In general population, PRS on eGFR was associated with incident CKD and kidney failure in the Atherosclerosis Risk in Communities study with 8.6% of the individuals having diabetes (146). In diabetes, smaller studies have shown that genetic risk scores for DKD improved the prediction of DKD in Han Chinese with T2D (147). In the ADjuVANt Chemotherapy in the Elderly (ADVANCE) trial with individuals with T2D, a multi-phenotype PRS, based on variants from the general population GWAS, predicted micro- and macrovascular complications and suggested that the PRS can identify high-risk individuals, who would benefit from intensified diabetes treatment (148); similarly, a general population PRS for coronary artery disease (CAD) was associated with CAD also among individuals with T1D (149). However, no large-scale PRS for DKD have yet been published, and larger GWASs on DKD are needed to create diabetes-specific PRS for DKD and to assess their utility compared to general population PRS.

To date, several CpG sites with altered methylation levels in DKD have been identified across the genome. Understanding the underlying mechanism behind these changes would be critical, i.e., are the observed changes driven by kidney disease or some other manifestation that emerges as the disease progress, and whether the changes are causal for the development or progression of DKD. In addition, methylation levels are also influenced by the genetics. Insights to the complex network behind the findings might therefore require integrating DNA methylation results with results from multiple other sources such as GWAS as well as transcriptomic and proteomic data. Some efforts in that direction have already been made. Indeed, a recent study demonstrated that DNA methylation explains a larger fraction of kidney disease heritability than gene expression by integrating GWAS data with methylomic and transcriptomic data obtained from 446 kidney tissue samples (88).

DNA methylation markers have proven useful for the prediction of DKD progression. Current studies, however, have focused on the later stages of kidney disease, when AER is severely increased or when kidney failure has occurred. EWASs at earlier stages of DKD, when AER is only moderately increased, could potentially identify additional CpGs and perhaps even more importantly, enable the prediction of early changes using DNA methylation. Although DNA methylation scores have not yet been as extensively implemented in risk prediction as the PRSs, methylation scores show a great promise as they incorporate information from both the genes and the environment. In a recent study, methylation scores improved the prediction of a range of clinical diagnoses and traits, including kidney disease, outperforming the predictive ability of polygenetic risk scores (140). However, the dynamic nature of methylation as well as its tissue-specificity introduces limitations regarding causality, time span of effect, and target tissue. By incorporating genetic information, causality can be addressed, and future studies may also be facilitated by emerging single-cell sequencing technologies that enable more targeted analyses, such as exploring the causal effects of DNA methylation at the single-cell level in the kidneys.

## Author contributions

NS and ED revised the literature and wrote the manuscript. P-HG critically revised the manuscript for the scientific content. All authors agree to be accountable for the content of the work. All authors contributed to the article and approved the submitted version.
